# Morphological transparency and markedness matter in heritage speaker gender processing: an EEG study

**DOI:** 10.3389/fpsyg.2023.1114464

**Published:** 2023-06-12

**Authors:** Alicia Luque, Eleonora Rossi, Maki Kubota, Megan Nakamura, César Rosales, Cristina López-Rojas, Yulia Rodina, Jason Rothman

**Affiliations:** ^1^Department of Applied Language Studies, Nebrija University, Madrid, Spain; ^2^Nebrija Research Center in Cognition, Nebrija University, Madrid, Spain; ^3^Department of Linguistics, University of Florida, Gainsville, FL, United States; ^4^Department of Language and Culture, UiT the Arctic University of Norway, Tromsø, Norway; ^5^Mind, Brain and Behavior Research Center, University of Granada, Granada, Spain

**Keywords:** heritage bilingualism, Spanish as a heritage language, grammatical processing, gender agreement, morhological transparency, morphological markedness, event-related potentials

## Abstract

The present study investigated the qualitative nature of grammatical gender knowledge and processing in heritage speakers (HSs) of Spanish living in the United States. Forty-four adult Spanish HS bilinguals participated, completing a behavioral grammatical gender assignment task and a grammaticality judgment task (GJT) while their brain activity was recorded using electroencephalography (EEG). The EEG GJT task included grammatical and ungrammatical sentences with grammatical gender violations on inanimate nouns, where transparency of the morpho(phono)logical cue and markedness were manipulated. The results of this study revealed that grammatical gender violations elicited the typical P600 effect across all relevant conditions, indicating that the grammatical representations and processing of grammatical gender in HSs are qualitatively similar to those in Spanish-dominant native speakers. Given the experimental manipulation in this study, these findings also suggest that both morphological transparency and markedness play significant roles in how grammatical gender is processed. However, the results of this study differ from those reported in previous studies with Spanish-dominant native speakers, as the P600 effect found was accompanied by a biphasic N400 effect. This pattern of results is interpreted as further evidence that the bilingual experience of HSs modulates certain aspects of morphosyntactic processing, particularly conferring a greater reliance on morphology. Additionally, the results of this study highlight the importance of incorporating neurolinguistic online processing methods to better understand what underlies HS bilingual competence and processing outcomes.

## 1. Introduction

Heritage speaker bilinguals (HSs) are native, early bilinguals of a heritage language (HL). A language qualifies as a HL when it is spoken at home—often a minoritized language—yet is distinct from the majority language(s) spoken within the larger societal context (e.g., Rothman, 2009; Montrul, 2011; Polinsky, 2018). Like homeland native speakers, HSs acquire their HL as a first language (L1), early and naturalistically. Yet, HSs often acquire the heritage L1 in a context of significantly reduced input and/or opportunities over the lifespan to use and/or be trained in it. Thus, it is unsurprising that a substantial amount of research has documented significant differences between HSs and homeland native speakers (Montrul, 2016; Polinsky, 2018; Polinsky and Scontras, 2020) across a wide range of grammatical domains. Among these, a widely studied domain—its acquisition and processing—is grammatical gender. Relevant studies report varied results, ranging from HS performance similar to what would be expected of homeland natives to data suggesting qualitatively different gender systems in HLs (e.g., Gathercole, 2002; Gathercole and Thomas, 2005; Polinsky, 2008; Kupisch et al., 2013; Unsworth et al., 2014; Fuchs et al., 2015; Montrul, 2016; Rodina and Westergaard, 2017; Scontras et al., 2018; Di Pisa et al., 2022). Innovations with gender in HS comprehension, production and processing are perhaps surprising considering that, at least when transparency of the grammatical gender system is high as in Spanish, both (lexical) assignment and (syntactic) agreement have been shown to be acquired early by homeland native children. In fact, a series of studies have shown that mastery of gender marking on articles and adjectives in homeland native children reach target-like levels (at around 90%) around age 4 (Pérez-Pereira, 1991; López Ornat et al., 1994; Lew-Williams and Fernald, 2007; Arias-Trejo and Alva, 2013). That said, Spanish-speaking homeland native children are sensitive to the morphophonological form of the nouns and acquire the gender of transparent nouns somewhat earlier than that of opaque ones (*cf.* Sadek, 1975; Montrul, 2004; Gathercole et al., 2022).

Given its early acquired status, its robust frequency and its obligatory and salient nature—e.g., in Spanish, a plurality of nouns have reliable, transparent morphological exponents, matched in agreement across all elements in the determiner phrase pre- (articles/determiners) and post-nominally (adjectives)—it is not clear why grammatical gender should be an *a priori* vulnerable domain.

With few exceptions (e.g., Fuchs, 2021, 2022; Di Pisa et al., 2022), experimental evidence for grammatical gender development in heritage languages largely comes from studies using offline behavioral methods, such as spontaneous and elicited oral production and comprehension tasks. While these results demonstrate differences in HS performances in gender agreement from homeland natives, online research methods, although scarce by comparison, question any generalization regarding the vulnerability of gender in HL grammars, i.e., beyond lexical assignment. Studies employing neuroimaging techniques such as electroencephalography (EEG) with HSs are scarce, despite compelling reasons to promote their use (*cf.* Bayram et al., 2021).

EEG measures the summation of post-synaptic potentials generated from groups of neurons firing at the same time. This activity, although spontaneous and naturally occurring, also changes as a result of different cognitive, perceptual, or sensory demands. This makes it an excellent tool for understanding the neuronal basis of higher-order cognitive processes, such as, but not limited to, language processing. Although there are several types of analyses one can do with EEG to study bilingual language processing and related neurocognition (see Rossi et al., 2022), the most common in psycholinguistic research is to analyze the EEG signal in the time domain as Event-Related Potentials (ERPs) in order to extract neural responses to a specific event (stimuli) by averaging the time-locked signal over multiple experimental trials (Luck and Kappenman, 2011). ERPs are thus represented as waveform components of the signal at a precise time in response to a given stimulus. In the case of language, although not specific to linguistic processing *per se*, components like the N400 or P600 reliably emerge, corresponding to matched language stimuli that do and do not contain specific types of anomalies (e.g., grammatical error, infelicity). Online methods are, in principle, less subject to issues of metalinguistic and literacy effects that have been argued to disproportionately affect HSs’ performances (Kupisch and Rothman, 2016; Polinsky, 2018). As such, examining how ERP components manifest while HSs process gender errors can offer unique insights into how their grammars are underlyingly represented and how such knowledge is deployed for processing beyond what can be understood from behavioral methods alone. Recent work using eye-tracking and self-paced reading for grammatical gender already suggests that HS processing is much less distinct from homeland natives than one might have expected from previous behavioral studies (e.g., Fuchs, 2021, 2022; Di Pisa et al., 2022). Thus, EEG promises to at least complement, if not go beyond, such evidence, allowing a look into how HSs’ brains process gender in real-time. With this in mind, the present study aims to fill several gaps simultaneously.

It is important to note that very few previous studies have used EEG to investigate HS linguistic processing (e.g., Van Rijswijk, 2016; Martohardjono et al., 2017). Given this, in the present study, we chose to venture into EEG with HSs within an otherwise well-studied domain of grammar in HSs, namely grammatical gender, using behavioral methods. Crucially, we do so against a backdrop of well-established use of EEG to examine gender processing for other relevant populations, namely functional monolinguals (i.e., in our terminology, homeland natives) and sequential second language (L2) bilinguals of Spanish. Using EEG with HSs, then, responsibly adds a new and crucial type of data to discussions that have emerged based on inconsistency in the HS behavioral literature of grammatical gender. Given that EEG can be an asset for adjudicating between previous ambiguous or contradictory data due to its high temporal resolution for capturing language processing in real-time, the relationship we assume between grammatical representations and real-time processing (Phillips and Ehrenhofer, 2015), and the fact that automatic brain responses are unlikely to be (less) subject to meta−/extralinguistic processes that could complicate (interpretations of) HSs’ empirical performances. At the same time, data from the present study can provide a test case on the efficacy of a largely absent source of evidence for HS processing more generally (*cf.* Bayram et al., 2021).

### 1.1. Grammatical gender system in Spanish

Grammatical gender (henceforth, gender) is an inherent property of nouns. Cross-linguistically, languages differ in terms of whether they have gender, and for those that do, the specificity of their particular system sits across at least two axioms: quantity and transparency. Whereas some languages have two genders, like Spanish, others have three or more (e.g., German). Yet not all so-called simple systems are equal, for example, while Spanish and Dutch each have only two gender values (masculine/feminine and common/neuter, respectively), there are important differences between the two. For instance, Spanish features a relatively transparent gender system, characterized by highly reliable morphophonological cues that indicate gender assignment. In contrast, Dutch presents a more opaque system. Nevertheless, regardless of the system’s relative transparency, certain patterns can be observed in how gender is generally assigned to nouns. These patterns include natural gender correspondence, as well as cues based on a word’s semantics or phonology. The latter becomes particularly apparent when examining inanimate nouns. However, gender assignment is generally arbitrary, with gender being reflected through syntactic agreement with other accompanying elements at the sentence level (Corbett, 1991).

Most Spanish dialects have a two-way gender system in which nouns are assigned either masculine or feminine values. Spanish nouns are marked for lexical gender using both transparent and opaque morphology. Transparent gender—where the final vowel reliably provides a cue to gender assignment—is signaled by the endings *-o* (*verano*_masc_ “summer”) or *-a* (*casa*_fem_ “house”) and is present in two-thirds of the Spanish lexicon (Harris, 1991). Indeed, approximately 99.5% of Spanish nouns ending in *-o* are masculine and around 96.3% of nouns ending in *-a* are feminine (Teschner and Russell, 1984). The remaining one-third of nouns in the Spanish lexicon do not offer strong distributional cues favoring one or the other gender assignment, except for those that offer other reliable gender cue patterns such as for the endings -*ción* and -*idad* in the case of feminine nouns. There are additional opaque gender cue patterns and tendencies which are also the focus of the current study, such as nouns ending in either a consonant (*pan*_masc_ “bread”_;_ *amistad*_fem_ “friendship”) or the vowel -e (*coche*_masc_ “car”_;_ *calle*_fem_“street”), which can be either masculine and feminine to similar degrees.

Additionally, current trends in morphological theory posit that Spanish masculine and feminine agreement features are asymmetrically represented (*cf.* Battistella, 1990; Harris, 1991; Cowper, 2005). Specifically, masculine is argued to be the default and thus unmarked relative to feminine. Under some approaches (e.g., Harris, 1991), masculine is actually the absence of a gender specification whereas feminine is the specific form that carries gender features. This approach would account for the fact that masculine is generally more frequent (new lexical entries to Spanish almost invariably take masculine), less error-prone in gender assignment, and the processing of agreement errors is often less costly than for feminine ones. Empirical work supports this position. For example, a corpus study by De la Cruz Cabanillas et al. (2007) revealed that 81.84% of English loanwords in Spanish were assigned masculine gender. Antón-Méndez et al. (2002) investigated noun-adjective gender agreement relations in homeland Spanish natives, finding that agreement errors were more frequent when the head noun was feminine (i.e., marked). Alemán Bañón and Rothman (2016) used EEG to show that agreement violations on marked elements are detected more easily. These findings are consistent with the claim that marked features are more disruptive to process.

### 1.2. Grammatical gender acquisition/processing

Regarding the acquisition of gender, research has shown that child HSs of Spanish achieve target-like mastery of gender at an early age (Pérez-Pereira, 1991; López-Ornat, 1997; Mariscal, 2009), not differing from what would be expected of milestones in homeland natives. By contrast, some longitudinal data from HS preschoolers acquiring Spanish in the US show that gender marking on articles and adjectives does not always reach ceiling accuracy by age 4 (Anderson, 1999). In fact, in some cases, Anderson’s study showed that gender errors persist and actually increase over time due to more exposure to the majority language (in this case English, a non-gender language). Errors are mainly attributed to the overuse of masculine with feminine nouns, an error pattern also reported for adult Spanish HSs (Lipski, 1993; Montrul et al., 2008; Hurr et al., 2020), more specifically, Montrul et al. (2008) showed that feminine gender was more “vulnerable,” especially with morphologically opaque nouns, as assessed by HSs’ performance in an oral picture description task.

While the picture emerging from behavioral tasks would suggest differences in HS Spanish gender systems compared to homeland natives, it is relevant to note that the degree of divergence is modulated by the modality of the experimental task, with oral tasks eliciting fewer errors than written tasks (*cf.* Montrul et al., 2008; Alarcón, 2011). For example, participants in Montrul et al. (2008) (*n* = 69, mean age = 22.7) produced on average 11% errors in an oral picture description task but 15 and 17% errors in a written picture interpretation and a written gender recognition task, respectively. Modality differences like this are not surprising, given that unlike homeland natives (or non-sequential L2 bilinguals for that matter), for HSs oral communication is not only by far the primary locus of language use, but in some cases, it is the only form. In sum, in the aggregate, evidence from offline behavioral studies with adult HSs of Spanish suggests that grammatical gender may be vulnerable in Heritage Spanish with gender transparency on the head noun being particularly error-prone for morphologically opaque feminine nouns.

Gender retrieval and agreement processing have, in general, been studied online rather extensively via eye movements (eye-tracking) and EEG (see Molinaro et al., 2011; Kaan et al., 2021 for review). However, besides a handful of recent studies using either self-paced reading/listening and eye-tracking, there is comparatively little available for (Spanish) HSs, and none using EEG. An eye-tracking study by Fuchs (2021, 2022) compared the use of gender predictively in the visual world paradigm in adult Spanish HSs (*n* = 21, mean age 22.3) and a group of homeland Spanish natives. The results demonstrate that HSs make use of the definite articles *el_masc_* and *la_fem_* to predict the gender of an upcoming noun in a manner qualitatively similar to homeland natives. Not surprisingly, some differences between the two groups still occurred. After all, the groups are in many ways not comparable, given important differences in their experiences with Spanish (see Rothman et al., 2022). Although HSs fixated on target nouns faster in gender mismatch than in match conditions, they were slower than the homeland natives in both conditions overall. Notwithstanding, the differences Fuchs reports are quantitative in nature, suggesting both groups have qualitatively similar gender representations.

A similar picture emerges from a recent processing study examining the role of morphological markedness in HL gender processing using a combination of online and offline measures, such as a self-paced reading task and a GJT, by Di Pisa et al. (2022). Although the HL in this study is Italian, the results complement Fuchs’ nicely and are of particular interest given what the present study examines. The Italian HSs showed clear evidence of a qualitatively similar underlying system of grammatical gender compared to homeland Italian natives. Moreover, the results from Di Pisa et al. (2022) also indicate a considerable modulatory role of gender transparency on the head noun as well as a markedness effect pertaining to the type of agreement error: feature clash errors were more costly than default ones. This pattern, only shown by the HS group, lead the authors to argue for a heightened dependency on overt morphology in the case of HS processing.

As mentioned, ERP research on HS gender processing simply does not exist, however, there is a substantial body of research on homeland Spanish natives and L2 learners of Spanish that, given the context of the present study, is worth briefly reviewing. Those studies have mostly focused on grammatical gender processing under conditions of agreement violations with transparent nouns (those ending in *-o* or *-a*). In their aggregate, findings from Spanish functional monolinguals convincingly show that determiner-noun agreement violations elicit a greater posterior positivity around 600 milliseconds (ms) after stimulus onset (P600), as compared to conditions without violations (Barber and Carreiras, 2005; Caffarra and Barber, 2015). The P600 effect has been argued to reflect processes of syntactic integration, reanalysis and repair (Osterhout and Mobley, 1995; O'Rourke and Van Petten, 2011), or non-syntactic late integration (Brouwer et al., 2012), as well as costs associated with structure building, checking and reprocessing (Van de Meerendonk et al., 2009). The typical P600 effect found in this domain can also (but not always) be preceded by an increased left anterior negativity (LAN) between 300 ms and 500 ms poststimulus (e.g., Barber and Carreiras, 2005), attributed to processes of automatic detection of morphosyntactic violations (Friederici, 2002), difficulties integrating mismatching information (Gunter et al., 2000), or working memory costs (Coulson et al., 1998).

Few ERP studies have compared how gender agreement violations with morphologically transparent vs. opaque nouns are processed and even fewer where morphological markedness is jointly or independently considered. For Spanish functional monolinguals (Caffarra and Barber, 2015), the LAN-P600 pattern has been observed for gender violations with both transparent and opaque nouns. Transparent nouns, however, elicited a greater LAN than opaque ones around 400 ms after the nouns. Yet, no interaction was found between the biphasic pattern and noun transparency. These results were interpreted as suggesting that functional Spanish monolinguals are sensitive to the formal gender cues on the nouns, but this distributional information does not have a strong impact on agreement computation. In other words, gender cues may be redundant in recovering gender and computing agreement dependences, at least for homeland-dominant speakers. A further comparison with two groups of Spanish-Basque early bilinguals by Caffarra et al. (2017) is of relevance, especially for the present study. This study tested Basque-dominant bilinguals and Spanish-dominant bilinguals in the Basque country, a bilingual region in Northern Spain. This study tested Basque-dominant bilinguals and Spanish-dominant bilinguals in the Basque country, a bilingual region in Northern Spain. The ERP results showed that dominant Basque bilinguals elicited only a P600 effect for gender violations on opaque nouns, whereas the Spanish-dominant bilinguals showed a pattern similar to the Spanish functional monolinguals in Caffarra and Barber (2015) i.e., a biphasic LAN-P600 effect. The authors conclude that the processing of gender violations with opaque nouns in particular is affected by potentially unstable lexical representations arising on a continuum dependent on the individual’s context of bilingualism and its ensuing reduction of experience with/use of Spanish on a daily basis. This is interesting in light of the behavioral evidence from the Basque-dominant bilinguals, those with the higher tendency to show the aforementioned effects, which showed high accuracy in online grammaticality judgment and an offline gender decision task. Such a result dovetails, in our view, nicely with the argumentation of Di Pisa et al. (2022), who interpreted their reaction time results also showing a transparency effect to indicate a greater reliance/awareness of bilinguals to overt morphological exponents. The fact that this only appears to be supported in the behavioral results, however, does not entirely offer clarity on the matter but might have something to do with differences in bilingualism contexts given that the Caffarra et al. (2017) bilinguals are not HSs and live in a context where naturalistic exposure to Spanish is omnipresent in all aspects of a bilingual society.

Among the EEG studies that have examined the role of morphological markedness for Spanish gender processing during online sentence comprehension, Alemán Bañón and Rothman (2016) investigated homeland native speakers’ processing and neural sensitivity to gender agreement violations in noun-adjective concord at a distance (with an intervening CP), where half of the nouns were masculine opaque (e.g., pastel_masc_ *“cake”*) and the other half were feminine opaque (catedral_fem_ *“cathedral”*). Results from their study showed that homeland Spanish natives elicited a P600 effect, suggesting that they were sensitive to agreement violations. They also suggested that morphological markedness modulates the magnitude of the effect: there was a significant difference both in the timing and amplitude of the P600 response to feature-clash violations as compared to the default ones. In other words, homeland Spanish natives detected and revised mismatching noun-adjective gender violations for feminine adjectives more quickly than for masculine ones. These results are consistent with previous studies relating the time course of the P600 with the detection of structural anomalies during sentence processing (*cf.* Sassenhagen and Bornkessel-Schlesewsky, 2015). Alemán Bañón et al. (2017) conducted the same experiment with Spanish L2 learners. The L2 learners, similar to the homeland Spanish natives in the Alemán Bañón and Rothman’s (2016) study, were sensitive to agreement violations as revealed by a P600 effect. This is especially noteworthy considering that the opaque morphological nature of the nouns in the experiment did not provide strong (morphophonological) distributional cues to gender. Additionally, the EEG data revealed that markedness also impacted online grammatical processing—a significantly earlier P600 effect emerged for feature-clash than default gender violation errors—although the effect was quantitatively smaller than for the homeland natives. On the behavioral side and potentially relevant for the context of HL processing, the results also indicated that the L2 participants made significantly more assignment than agreement errors, suggesting that L2 bilinguals had less difficulty with the syntactic aspects of gender than the lexical ones.

### 1.3. Research questions and hypotheses

With the contexts provided in this background review, we pose the following questions and hypotheses:

Question 1: What are the event-related potential (ERP) signatures of grammatical gender agreement processing in Spanish as a Heritage Language?

Based on previous research, we expect to find qualitatively similar effects in HSs for grammatical gender processing as has been reported in the literature for other native speakers of Spanish. In other words, we expect to at least see evidence of a P600 effect. In line with the results by Caffarra et al. (2017), we do not expect a LAN to accompany the P600 precisely because our HSs of Spanish are English-dominant speakers who are likely to have significantly less use of /exposure to Spanish—at least at the aggregate level—than the Basque-dominant Spanish speakers, who did not show a LAN effect.

Question 2: Do we find evidence of neurophysiological signatures related to the processing of grammatical gender being modulated by various aspects of overt morphology (i.e., transparency and markedness)?

Following from what Di Pisa et al. (2022) argue, if it is the case that HSs are more reliant on overt morphology—even when in Caffarra and colleagues’ words it is redundant as is the case with gender agreement in Romance languages—we would expect our HSs to be highly sensitive to both transparency and markedness. Note, however, that the two sit at various levels of complexity. This could play out differentially for HSs even if the general proposal that they are more sensitive to morphology is on the right track: transparency sits at the level of the lexical representation of individual nouns whereas markedness characterizes the gender system itself. As such, all things being equal, we would expect markedness to robustly affect HS processing across the board. We expect this to be reflected via differences in the amplitude of the ERP signatures reflecting the relative cost of processing a default error over a feature- clash one (see Alemán Bañón and Rothman, 2016), due to HSs’ enhanced morphological sensitivity, potentially bootstrapped by a more generalized HS reliance on defaults overall (Polinsky, 2018). Alternatively, while we expect potential transparency effects, as they might be modulated by other individual factors distinguishing HSs from each other (e.g., HL proficiency or use/exposure) this effect is more likely to be washed out in an aggregated analysis.

## 2. Methods

### 2.1. Participants

Given the rich EEG literature on grammatical gender processing in Spanish for homeland natives and successive L2 bilinguals, from which we have established EEG signatures for the experimental stimuli we use, and following the argumentation of Rothman et al. (2022) that questions the need, utility and appropriateness of monolingual comparison groups under such circumstances, our population herein is solely comprised of HSs: 44 (32 = females) English-dominant HSs of Spanish. At the time of testing, all participants were enrolled as undergraduate students at the University of Florida in the US. All our HS participants reported being native speakers of Spanish and having acquired English simultaneously or sequentially in childhood as an L2. Additionally, 4 participants reported being native speakers of (heritage) Portuguese.^1^ The criteria to participate in the study required individuals to indicate via a pre-screening questionnaire that they (a) had been exposed to Spanish either at home or in the community before age 5, (b) to have normal or corrected-to-normal vision and hearing, be right-handed, and (c) to have no history of diagnosis of neurological or learning disorders. See Table 1 for demographic details characterizing our participants, including scores for key measures we detail in the following section.

**Table 1:** Participant characteristics.

	*M* (SD) [Range]
Sex	31 females
Age (years)	20.02 (1.49) [18–24]
Number of native languages^a^	2.06 (0.26) [2–3]
Number of additional languages^b^	0.42 (0.76) [0–3]
Spanish: Age of first exposure^c^ (years)	0.98 (0.95) [0–5]
English: Age of first exposure^c^ (years)	3.7 (2.54) [0–10]
Spanish: Percentage of daily social language use^d^	11.86 (13.51) [0–80]
English: Percentage of daily social language use^d^	70.31 (35.08) [13.33–100]
Spanish: Self-rated listening proficiency^e^	6.65 (0.65) [4–7]
Spanish: Self-rated speaking proficiency^e^	6.11 (0.85) [4–7]
Spanish: Self-rated writing proficiency^e^	5.32 (1.12) [3–7]
Spanish: Self-rated reading proficiency^e^	5.86 (1.01) [3–7]
English: Self-rated listening proficiency^e^	6.74 (1.09) [6–7]
English: Self-rated speaking proficiency^e^	6.69 (1.10) [6–7]
English: Self-rated writing proficiency^e^	6.46 (1.20) [4–7]
English: Self-rated reading proficiency^e^	6.67 (1.10) [6–7]
LexTALE-Span^f^	59.3 (6.60) [50–74]

a Including Spanish and English. Additionally, 4 participants reported being also native speakers of (heritage) Portuguese.

b Including Portuguese, French, German, Mandarin Chinese, Italian, Bengali, Russian, Korean, Japanese, and American Sign Language (ASL).

c Due to the fact that all participants indicated having been exposed to both languages before the age of 5, these answers respond to the following question: “When did you start using language Spanish/English at home or at school (whichever came first)”?

d Based off participants’ responses to how many hours a day they spent talking to non-family members (i.e., friends, co-workers, other). We took 15 h/day to represent 100% given that we are supposed to sleep 8 to 9 h on average, thus, if a participant reported spending a total of 6 h a day speaking English or Spanish to non-family members, we considered that to represent 40% of their percentage of daily social language use (e.g., (6×100)/15 = 40%).

e Self-rated proficiency on 1 (‘Very Poor) to 7 (‘Excellent) scale.

f LexTALE-Span= Lexical Test for Advanced Learners of Spanish. The original version of the task consists of a total of 90 items (Izura et al., 2014). However, due to a technical issue during task administration, some of our participants were only presented with 87 items. To maintain consistency in our group results analysis, we adjusted the total number of items to 87 for all participants. The score reported here represents the averaged percentage of accurate responses in the task.

### 2.2. General study design

The present study is part of a larger study; in this section, we only report the details regarding the tasks specifically related to examining grammatical gender agreement processing in Spanish as a HL. The present study was comprised of a pre-screening and one in-lab experimental session. During the pre-screening, participants provided informed consent and completed an online questionnaire, aimed at gathering detailed language and demographic history background information using the LHQ.3 (Li et al., 2020), as well as general health and handedness. Participants meeting the pre-screening criteria were invited to the in-lab experimental session. For this session, all in-task instructions were written in Spanish. First, participants completed a lexical decision task in Spanish (LexTALE-Span; Izura et al., 2014) as an objective proficiency measure and a Spanish gender assignment task—testing each participant’s assigned gender value for the full set of nouns used in the EEG experiment. For both the lexical decision and the gender assignment task, response accuracy and RTs were collected. After these behavioral measures were complete, participants were fitted with an EEG and sat for the capping procedure for approximately 15–20 mins. Lastly, participants completed the main task, a Spanish grammaticality judgment task (GJT), while EEG was recorded. Upon completion of the study, participants were then debriefed and compensated with either course credit or a $40 gift card.

### 2.3. Materials and procedure

#### 2.3.1. Gender assignment task

In order to obtain each participant’s own baseline for lexical gender assignment, the gender assignment task used the same set of nouns that would be presented in the sentences in the GJT. Three versions were created and counterbalanced across participants; a participant assigned to version 1 of the gender assignment task also completed version 1 of the GJT. Thus, each participant saw a total of 180 critical nouns, 90 masculine inanimate nouns (30 transparent and 60 opaque) and 90 inanimate feminine nouns (30 transparent and 60 opaque). A total of three blocks were created comprised of 30 items each. Words in each block were automatically randomized. Participants were seated in front of a 22-inch monitor. The task was presented in E-Prime 3.0 (Psychology Software Tools, 2016, Pittsburgh, PA) and completed on a computer using a keyboard. Participants were asked to indicate the grammatical gender of each word presented in the screen by selecting the appropriate gender-marked determiner from two options *el*_masc_ or *la*_fem_ appearing on the screen. During each trial, a fixation cross was presented for 500 ms. Then, each word appeared in the middle of the screen for 500 ms. After the word was presented, a prompt indicated that a response was required. The next trial began following their response. The task took approximately 7 mins to complete.

#### 2.3.2. Grammaticality judgment task

The EEG GJT had a 2x2x2 design with grammaticality (grammatical vs. ungrammatical agreement), gender (masculine vs. feminine) and transparency (transparent vs. opaque) as factors. Each condition consisted of 60 sentences with grammatical agreement targeted at the adjective, resulting in a total of 240 grammatical sentences. Another set of 60 sentences for each condition type was created by manipulating ungrammatical gender agreement between the target noun and its corresponding adjective across the four experimental conditions, resulting in a total of 240 ungrammatical sentences, 60 per experimental condition. Each of the four experimental conditions included grammatical and ungrammatical items for each gender, half of the critical inanimate nouns had transparent endings (masculine *-o* and feminine *-a*) while the other half had opaque endings (−*e* or consonant). Even though we tried to control for frequency as closely as possible, given the attested differences in frequency between masculine vs. feminine and transparent vs. opaque, in our study there was a significant difference in log frequency (based on the SUBTLEX-ESP corpus, Cuetos et al., 2012) between masculine and feminine nouns (*t* = −2.33 *p* = 0.02) as well as between transparent and opaque nouns (*t* = −2.97, *p* = 0.003), as expected. To account for this, we included frequency as a control variable in the behavioral accuracy model (as described in 3.2.1). These 480 sentences were counterbalanced across three experimental lists, such that a given learner would see a total of 40 items per condition (20 grammatical and 20 ungrammatical) for each of the four experimental conditions (i.e., masculine transparent, masculine opaque, feminine transparent, feminine opaque). Importantly, no participant saw the same sentence twice. Markedness was also manipulated within the ungrammatical agreement conditions via directionality of the overt marking on the adjective concord: (a) default errors had a feminine noun with a masculine inflected adjective and (b) feature clash errors had a masculine noun with an adjective inflected as feminine. Importantly, we made sure that grammatical gender only appeared as a morphosyntactic feature without any semantic significance. In other words, all items that were included as part of the relevant gender conditions in the present study had grammatical gender, but no semantic or natural gender (assigned based on the semantic notion of biological sex; see Table 2).

**Table 2:** Example grammaticality judgment task stimuli by condition.

Condition	Grammatical control	Ungrammatical violation
Masculine Transparent	Mateo visitó un_masc_ pueblo_masc_ *pequeño*_masc_ con sus amigas. Mateo visited a_masc_ *small*_masc_ town_masc_ with his friends.	*Mateo visitó un_masc_ pueblo_masc_ *pequeña_fem_* con sus amigas. *Mateo visited a_masc_ *small_fem_* town_masc_ with his friends. *(feature-clash error)*
Masculine Opaque	Carla pidió un_masc_ postre_masc_ *dietético*_masc_ después del almuerzo. Carla ordered a_masc_ low-calorie_masc_ dessert_masc_ after lunch.	*Carla pidió un_masc_ postre_MASC_ *dietética*_fem_ después del almuerzo. *Carla ordered a_masc_ low-calorie_fem_ dessert_masc_ after lunch. *(feature-clash error)*
Feminine Transparent	Leonor vio una_fem_ película_fem_ *romántica*_fem_ en el cine. Leonor watched a_fem_ *romantic*_fem_ movie_fem_ in the theater.	*Leonor vio una_fem_ película_fem_ *romántico*_masc_ en el cine. *Leonor watched a_fem_ *romantic*_masc_ movie_fem_ in the theater. (*default error*)
Feminine Opaque	María dio una_fem_ clase_fem_ *entretenida*_fem_ el lunes pasado. María taught an_fem_ *engaging*_fem_ class_fem_ last Monday.	*María dio una_fem_ clase_fem_ *entretenido*_masc_ el lunes pasado. *María taught an_fem_ *engaging*_fem_ class_fem_ last Monday. (*default error*)

Italics indicate the critical word in each sentence. Violation sentences are indicated by an asterisk. As shown above, transparency is assessed via the potential differences between the transparent and opaque conditions, and markedness is assessed via the potential differences between the masculine and feminine ungrammatical agreement conditions.

Since Spanish requires the determiner to be present before the noun in all sentences with adjectival modifiers—bare nominals are impossible—we added an additional set of 60 ungrammatical sentences that contained agreement violations between the determiner and its head noun, i.e., *Mariano fotografió una_fem_ tornado_masc_ peligroso_masc_ (*Mariano photographed a_fem_ *dangerous*_masc_ *tornado_masc_)*. This was done simply to avoid the pattern that all sentences in the experiment provided the correct gender assignment cue via the pre-nominal article. Additionally, the GJT included 240 sentences containing number agreement violations that are part of a different study. Finally, an additional set of 120 filler items were included. For all sentences, length ranged from 7 to 8 words. None of the critical words were repeated, and violations never occurred in initial or final sentence positions. In sum, the GJT was comprised of a total of 900 sentences, however, in this manuscript we report only findings for trials including the gender agreement conditions described.

Like the assignment task, all experimental items were distributed across three lists using a Latin square design such that participants only viewed one sentence from each sentence frame. In total, each list contained 420 sentences (240 experimental items/180 filler items). A total of six blocks were created comprised of 70 items. Sentences in each block were automatically randomized.

**Figure 1:**
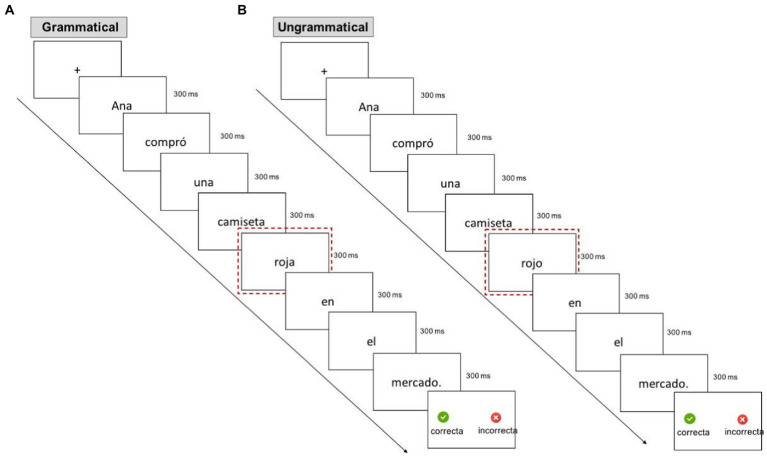
Schematic representation of example trial sequence from the grammaticality judgment task. **(A)** Illustrates the Grammatical condition and **(B)** the Ungrammatical condition. The dotted red element illustrates the target item.

Experimental sentences were presented using E-Prime 3.0 software in a rapid serial visual presentation (RSVP) paradigm. Participants read sentences in Spanish one word at a time in the center of the screen EEG was recorded and were instructed to indicate grammaticality at the end of each sentence via a button-press using an external keyboard. Each trial started with a 500 ms fixation cross followed by a 150 ms interstimulus interval (ISI). Then, each word appeared in the middle of the screen for 300 ms followed by a 150 ms ISI for all sentence items except for the last one. The next trial began following their responses. The task took approximately 50 mins to complete (Figure 1).

### 2.4. EEG recording and pre-processing

Continuous EEG data were acquired using an array of 32 Ag/AgCl scalp electrodes using BrainVision Products active electrodes (Brain Products GmbH, Gilching, Germany) organized in accordance with the 10–20 system. Additionally, vertical and horizontal eye movements were measured using two sets of bipolar electrooculogram (EOG) electrodes placed above and below the left eye (vertical) and on the right and left canthi (horizontal). An online reference electrode was placed on the right mastoid and another was placed on the left mastoid for later re-referencing. Impedances were maintained at <10 kΩ. The signal was amplified using a Brain Vision actiCHamp amplifier with a 24-bit analog to digital conversion and was continuously recorded at a 1,000 Hz sampling rate without online filters. All data were pre-processed offline using Brain Vision Analyzer version 2.2 (Brain Products GmbH, Gilching, Germany). All EEG data were re-referenced to the average of both mastoids and filtered using a 0.1–30 Hz IIR Butterworth filter with a 12 dB slope. An independent components analysis (ICA) was used to identify and remove vertical and horizontal eye movements. After ICA, the data were subjected to a final inspection. All final artifact rejection was done using a semi-automatic mode followed by visual confirmation. Participant data with artifact rejection rates greater than 25% were excluded from the analysis, resulting in the loss of 1 participant. Additionally, three more participants were excluded due to technical issues during EEG data acquisition. After excluding these participants, the overall mean rejection rate remained below 10%. The final analysis was conducted on correct responses only, with an average of included trials across participants of 30.21 (SD = 6.65), 27.53 (SD = 7.03), 28.97 (SD = 6.72), and 27.26 (SD = 7.20) in the masculine transparent, masculine opaque, feminine transparent, and feminine opaque conditions, respectively (out of a total of 40 trials each participant saw per experimental condition).

**Table 3:** EEG data analyses: summary of the Chi-square and the *p*-values of the main effect of grammaticality across each time-window.

Time window	Chisq	*p*-value
100 to 200 ms	0.64	0.42
200 to 300 ms	0.35	0.55
300 to 400 ms	0.75	0.38
400 to 500 ms	12.20	< 0.001**
500 to 600 ms	0.66	0.41
600 to 700 ms	0.51	0.47
700 to 800 ms	18.38	< 0.001**
800 to 900 ms	9.39	0.002*
900 to 950 ms	2.05	0.15

*p < 0.005.**p < 0.001 (Bonferroni correction applied).

### 2.5. ERP analysis

Once the pre-processing steps were complete, epochs were extracted, and baseline corrected across all trials and across all conditions from -200 ms to 0 ms then averaged by condition. Mean amplitude ERP data were analyzed in 100 ms moving windows beginning from 0 ms prior to stimulus onset to 950 ms post-onset. A total of 10 windows were extracted. All 10 extracted time windows were included in our analysis. Analyses were conducted only for correct trials. Given the exploratory nature of this study, we did not necessarily expect that HSs would evidence the same ERP components observed in the functional monolingual literature (P600 and possibly the N400 and LAN), however, we were guided by them. Thus, we decided to focus our analyses on the full-time spectrum to be able to capture, if present, the early and later ERP components that have been consistently shown with different aspects of grammatical gender processing. All stimuli, data, and analyses scripts can be found on the following public OSF repository.^2^

### 2.6. Statistical analyses

**Figure 2:**
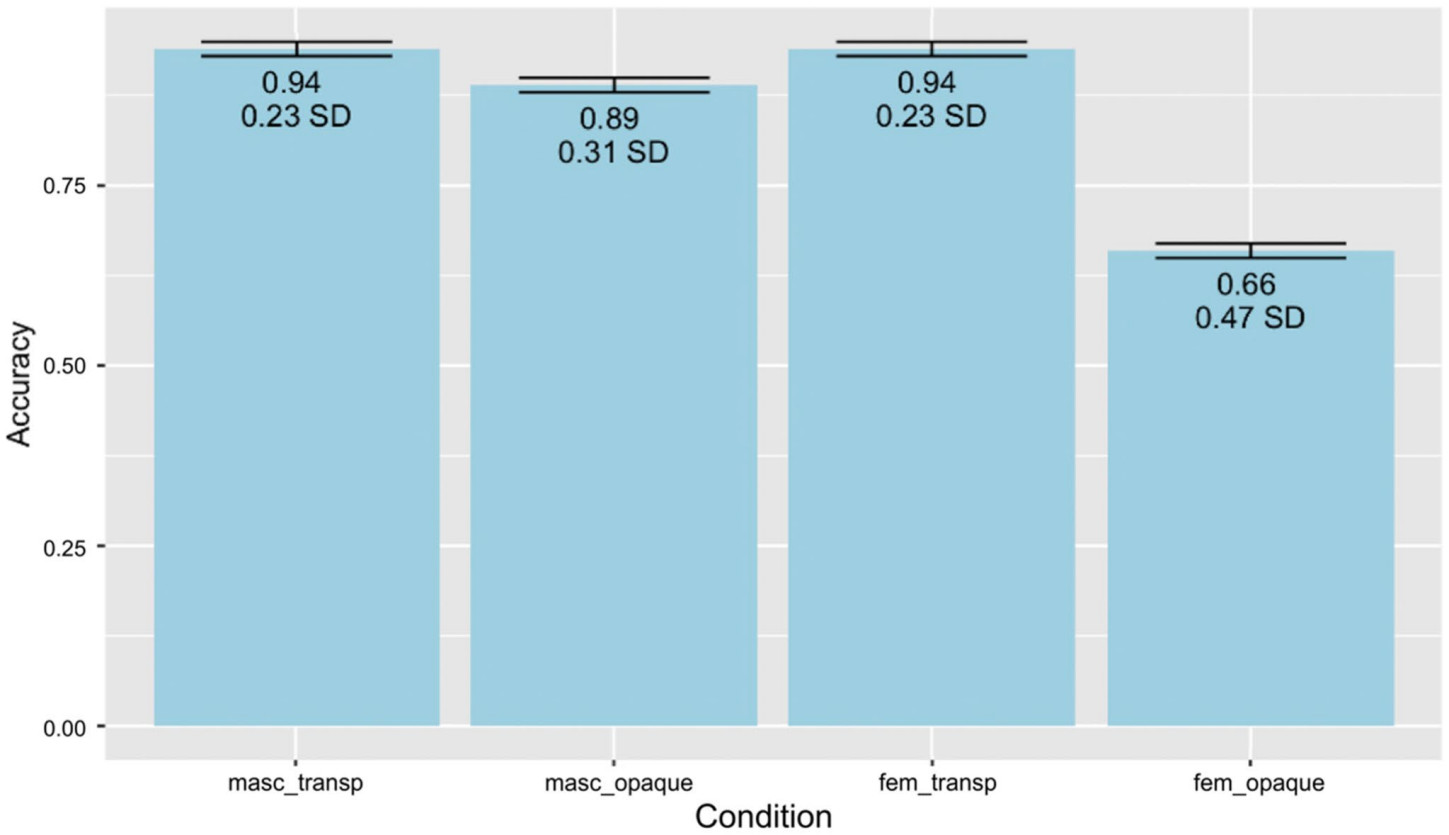
Gender assignment task: behavioral accuracy split by experimental condition. Error bars indicate standard error.

**Figure 3:**
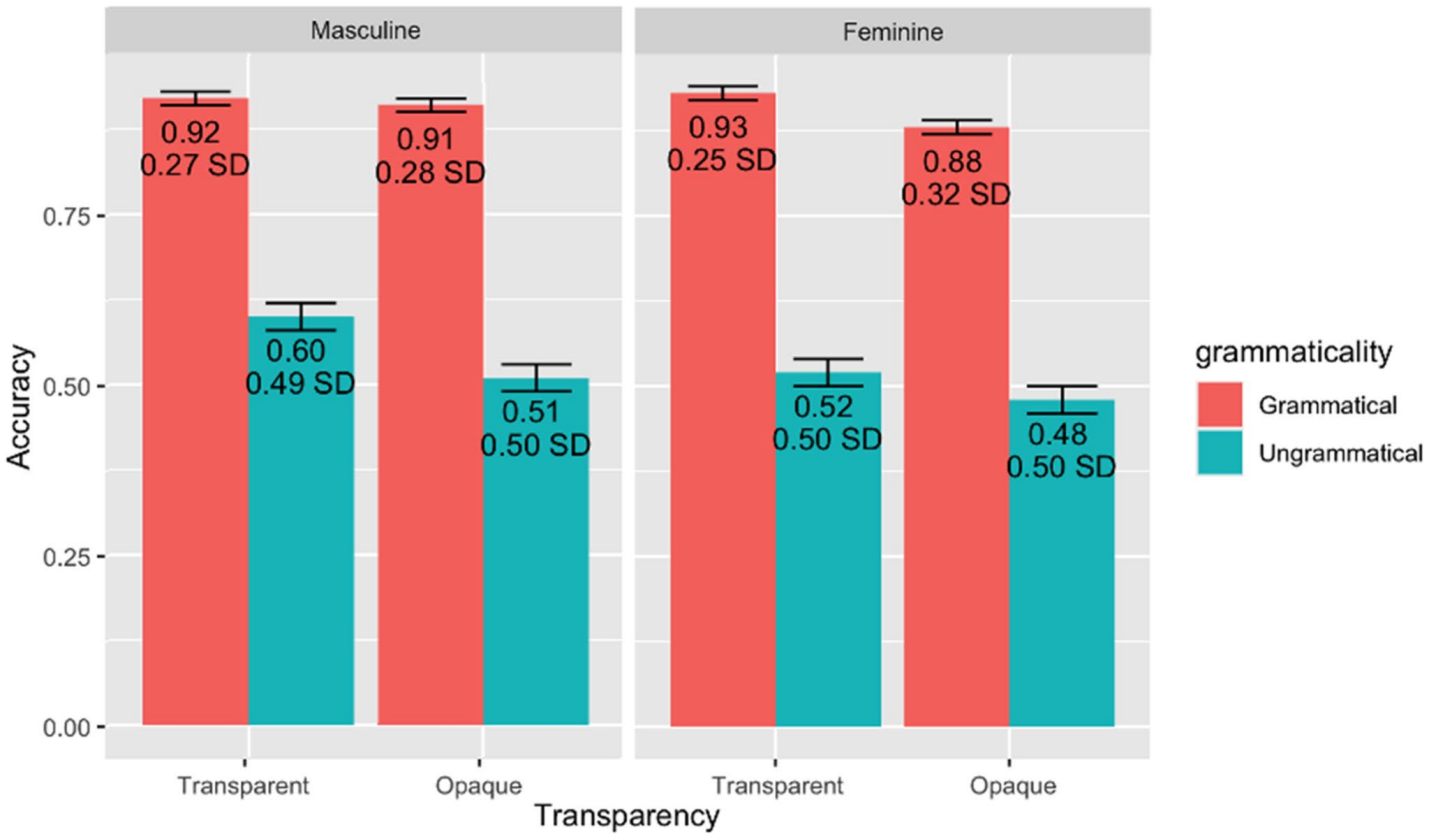
Grammaticality judgment task: behavioral results, split by gender, grammaticality, and transparency. Error bars indicate standard error.

**Figure 4:**
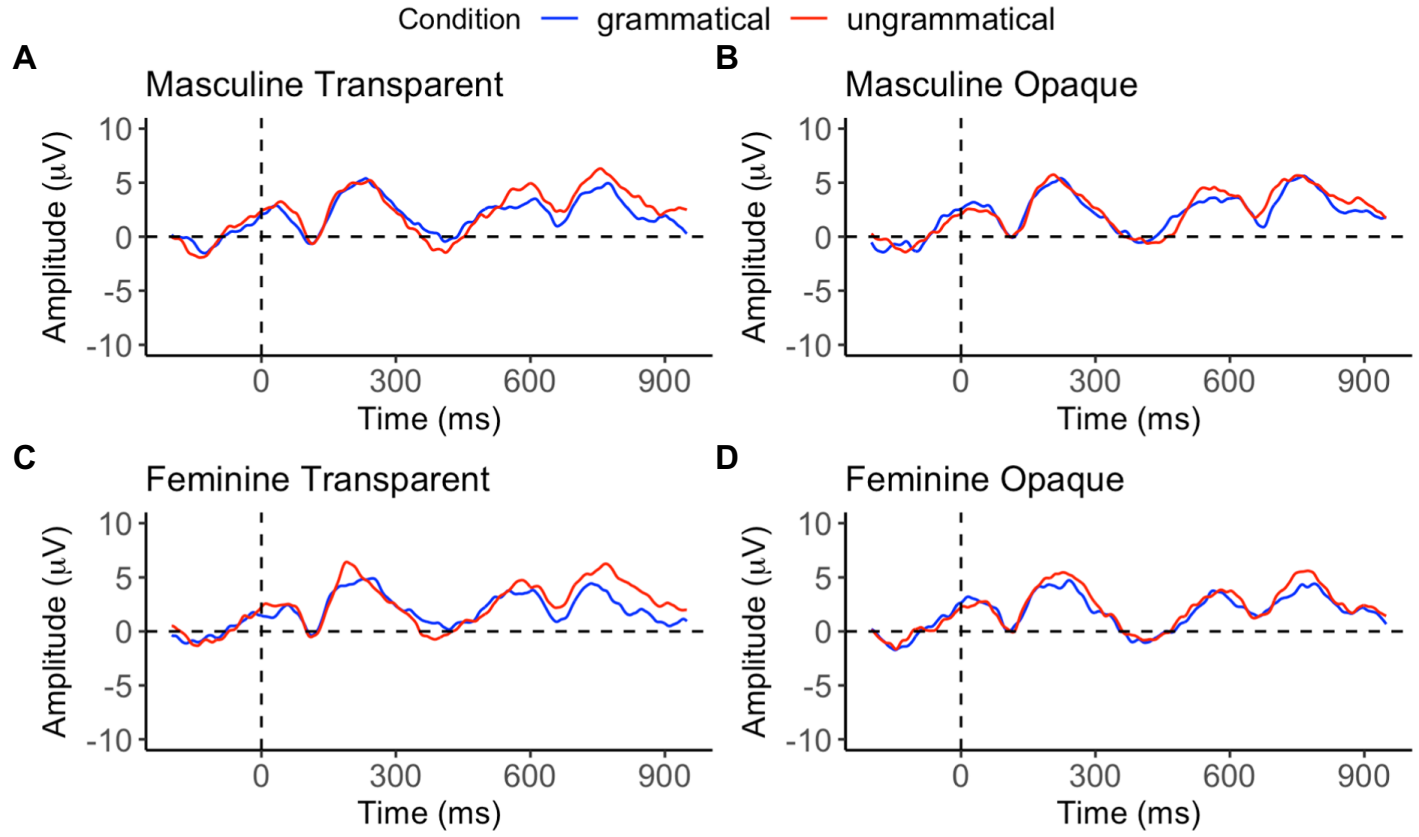
ERP waveforms across the transparent (**A**: masculine; **C**: feminine) and opaque (**B**: masculine; **D**: feminine) gender agreement conditions.

**Figure 5:**
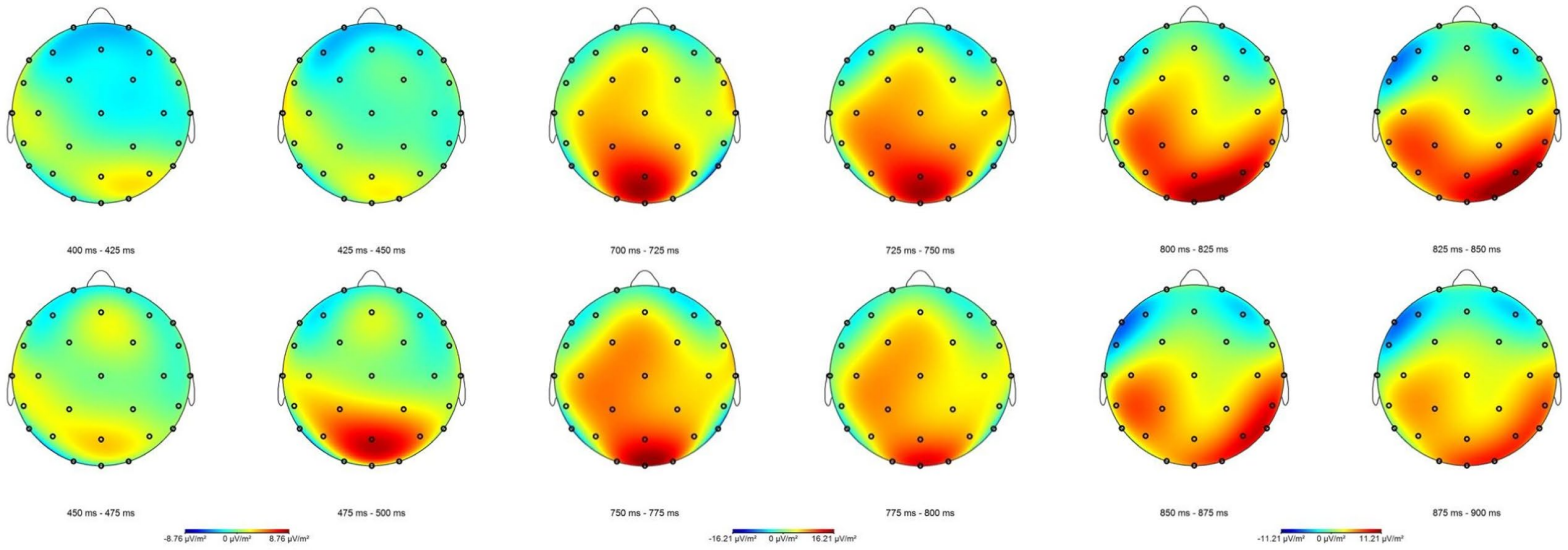
Topographic maps illustrating spatial distribution of the averaged brain responses elicited to the ungrammatical condition for the 400–500, 700–800, and 800–900 ms time-windows collapsed across the transparent/opaque and masculine/feminine conditions across all participants. It should be noted that while it is standard practice to use consistent scales when plotting scalp maps, we have intentionally employed different scales for each time window represented above. This decision was made to ensure a more representative portrayal of the effect distribution across the different time windows, considering their observed differences in magnitude. We acknowledge this deviation from conventional methodology but assert that by employing individualized scaling for each time window, we aim to provide a more accurate and visually representative depiction of the distribution of the effects found across the different time windows in order to offer a more informative and insightful visualization of the data.

Performance data from the gender assignment task and the behavioral and EEG portions of the GJT were analyzed using generalized linear mixed effects models (Baayen et al., 2008) in R (R Core Team, 2016). Pairwise *post-hoc* comparisons with Tukey’s contrasts were conducted using the *emmeans* package (Lenth, 2022). Additionally, likelihood ratio tests were conducted to analyze performance on the EEG portion of the GJT using the mixed function in the *afex* package (Singmann et al., 2022). All categorical variables were sum-coded and numerical variables were centered around the mean. The *ggplot2* package (Wickham, 2016) was used to generate Figures 2, 3, which illustrate performance (i.e., accuracy) on the gender assignment task and the behavioral portion of the EEG grammaticality judgment task. Additionally, ggplot2 was used to create Figure 4, showcasing the time course of group-averaged brain signatures associated with the processing of the experimental conditions under investigation. Brain Vision Analyzer 2.2 was employed to generate Figure 5, which displays the topographical distribution of the ERP effects found across the different time-windows explored.

## 3. Results

### 3.1. Gender assignment task

Descriptive results show higher accuracy for masculine than feminine (Masculine: *M* = 0.91, SD = 0.27, Feminine: *M* = 0.8, SD = 0.35) and for transparent over opaque conditions (Transparent: *M* = 0.94, SD = 0.23, Opaque: *M* = 0.77, SD = 0.39), with feminine opaque being the lowest overall. Overall accuracy of the gender assignment task is presented in Figure 2. The results of the generalized linear mixed effects model (Marginal *R*^2^ = 0.19; Conditional *R*^2^ = 0.38) further demonstrate a significant main effect of gender (*Chisq* = 74.29 *p* < 0.001), transparency (*Chisq* = 360.05, *p* < 0.001), as well as an interaction between gender and transparency (*Chisq* = 73.46, *p* < 0.001). This confirms that participants performed better on masculine than feminine (*E* = −0.41, *z* = −8.79) and on transparent than opaque (*E* = −0.80, *z* = −16.92) conditions. The only post-hoc comparison that was not significant was between feminine transparent and masculine transparent conditions (*E* = −0.003, *z* = −0.01, *p* = 1.00). In general, then, we can say that performance on the lexical gender assignment task for the nouns used in the EEG study indicates that participants performed at a rather target-like level. Not surprisingly, HSs’ assignment diverges from the gender values traditionally ascribed to particular nouns and occurs when the morphology does not offer direct cues, that is, for opaque nouns. Focusing on opaque nouns, we already note what seems to be a markedness effect whereby feminine assignment is significantly degraded with respect to masculine. This pattern can either reflect a true and direct markedness effect or result from an indirect markedness effect whereby a default assignment strategy of assigning masculine is utilized.

### 3.2. Grammaticality judgment task

#### 3.2.1. Behavioral results

Descriptive results demonstrate (on the aggregate level) that participants had higher accuracy on grammatical than ungrammatical items (Grammatical: *M* = 0.91, SD = 0.28, Ungrammatical: *M* = 0.53, SD = 0.53). Specifically, participants had higher accuracy for the masculine condition than the feminine one (Masculine: *M* = 0.74, SD = 0.44, Feminine: *M* = 0.70, SD = 0.46). Additionally, participants had higher accuracy for the transparent condition than the opaque one (Transparent: *M* = 0.74, SD = 0.44, Opaque: *M* = 0.70, SD = 0.46). Moreover, participants performed worst on the feminine opaque condition (see Figure 3 for overall accuracy on the behavioral portion of the GJT). The output from the generalized mixed effects model (Marginal *R*^2^ = 0.26; Conditional *R*^2^ = 0.52) corroborates the above-mentioned descriptive results by demonstrating a main effect of grammaticality (*Chisq* = 1589.36, *p* < 0.001), gender (*Chisq* = 6.39, *p* = 0.012), transparency (*Chisq* = 16.44, *p* < 0.001), and log frequency (*Chisq* = 11.69, *p* < 0.001) as well as a significant interaction between grammaticality, gender, and transparency (*Chisq* = 5.33, *p* = 0.021). The estimate of these results confirms that participants performed (a) better on grammatical than ungrammatical items (*E* = 1.32, *z* = 34.04), (b) better on masculine than feminine items (*E* = −0.11, *z* = −3.04) as well as (c) better on transparent than opaque items (*E* = −0.17, *z* = −4.76). The significant three-way interaction indicates that the difference in accuracy between grammatical and ungrammatical conditions were both modulated by gender and transparency, following the pattern of what behavioral studies with Spanish homeland natives have also reported (e.g., Pérez-Pereira, 1991; Afonso et al., 2014). Again, taken together, our results indicate a significant role of the morphophological exponents of gender at the levels of transparency as well as markedness. In other words, incorrect agreement on the adjective seems easier to judge when there is a clash between the default masculine feature of the head noun and the feminine feature of the adjective in general, and especially so when the head noun is marked with the transparent masculine ending *-o*.^3^

### 3.3. ERP results

EEG data were analyzed in two steps as follows: first, we ran a linear mixed effects model for each moving time window and included only grammaticality (grammatical, ungrammatical) as a fixed effect and subject and electrode as random intercepts. This first step was taken to explore the main ERP components that were elicited by the design. Performing this first step-model was important to identify the ERP signatures in response to the main manipulation (grammatical, ungrammatical) observable in HSs, who might have varied considerably from the ones reported for sequential L2 bilinguals or homeland natives. Recall that this is the first EEG study with Spanish HSs for this domain and given the fact that HSs have been shown to differ significantly from these other groups with respect to performance in behavioral tasks, we did not assume *a priori* that their brain responses would overlap with what has been shown for other groups of Spanish speakers, despite being guided by the components traditionally found within relevant previous studies with homeland natives. If the first model were to indicate a main effect of grammaticality (i.e., a significant amplitude difference between grammatical and ungrammatical conditions), as it did, a second linear mixed effects model would be performed (and was) including transparency (transparent, opaque) and gender (masculine, feminine) as well as interactions as fixed effects and subject and electrode as random intercepts. This measure was taken to examine whether transparency or gender (or their interaction) modulated the effect of the specific ERP components observed in the first step.

For the first linear mixed effects model (with Bonferroni correction), results show a significant main effect of grammaticality in the 400 to 500 ms (*E* = 0.12, *t* = 3.49), 700 to 800 ms (*E* = −0.19, *t* = −4.29), and 800 to 900 ms windows (*E* = −0.14, *t* = −3.06). With the exception of the 400 to 500 ms window, all estimates are positive, indicating that ungrammatical conditions elicited more positive amplitudes than the grammatical ones. In contrast, in the 400 to 500 ms window the estimates are negative, indicating that ungrammatical conditions elicited more negative amplitudes than grammatical conditions (see Table 3, for summary of results from first linear mixed effects model and see Figures 4, 5 for visual representation of the effects found). In sum, we find clear evidence of a P600 effect as found in the functional monolingual processing literature (and in some of the adult L2 literature as well). This alone demonstrates sensitivity to grammatical gender in a qualitatively similar way for the present HSs. However, unlike what has been found for homeland natives, the P600 here is accompanied by a clear N400 effect. The N400 effect has been traditionally argued to reflect lexical-semantic processing at the neural level, particularly semantic incongruency or the violation of lexical expectations, in the functionally monolingual processing literature (*cf.*, Kaan, 2007; Kutas and Federmeier, 2011).

While some studies with homeland Spanish natives show a LAN effect, the observed negativity in our data is topographically distributed over central electrodes (as seen in Figure 5) confirming its status as a genuine N400. It is not the case that the N400 has never been observed in Spanish gender processing studies. It has been noted, for example, with other sets of English-dominant bilinguals of Spanish, that is, with Spanish L2 learners (Gabriele et al., 2013), but this occurs when the L2 subjects are at low levels of proficiency and in the absence of any P600 signature. At higher levels of L2 Spanish proficiency, the reported N400 gets replaced by a P600, as shown nicely in the developmental work tracking adult L2 learners over time through the process of Spanish learning (Gabriele et al., 2013; Alemán Bañón et al., 2018). As such, the N400 at lower levels of L2 proficiency could be interpreted as a marker of development, indicating something qualitatively distinct in the processing of gender anomalies (i.e., the recognition of asymmetrical morphological patterns via matching) until reaching higher levels of proficiency where the syntax is in place. Given the high proficiency of our HSs as well as the P600 effect, we do not interpret the present N400 in the same way, a point to which we return downstream.

The results of the second linear mixed effects model (with Bonferroni correction) are provided in Table 4. In the 400 to 500 ms window, we found no significant two-way or three-way interaction, indicating that ungrammatical conditions elicit a greater negativity than grammatical conditions, regardless of transparency or gender. In the 700 to 800 ms window and 800 to 900 ms window, there was a significant two-way interaction between grammaticality and transparency as well as grammaticality and gender. Post-hoc comparisons revealed that (a) transparent conditions elicited greater positivity than opaque conditions (*p* < 0.001) and (b) masculine conditions also elicited greater positivity than feminine conditions (*p* < 0.001).

**Table 4:** EEG Data Analyses: Summary of Results from the Likelihood Ratio Test run as part of the linear mixed effects model across the 400 to 500 ms, 700 to 800 ms, and 800 to 900 ms time windows.

400 to 500 ms	Chisq	*p*-value
Grammaticality	12.12	<0.001**
Transparency	23.24	<0.001**
Gender	11.32	<0.001**
Grammaticality:transparency	0.70	0.40
Grammaticality:gender	1.45	0.22
Transparency:gender	3.80	0.051
Grammaticality:transparency:gender	0.21	0.65
*R*^2^ Marginal = 0.006; *R*^2^ Conditional = 0.13		

700 to 800 ms	Chisq	*p*-value
grammaticality	18.69	<0.001**
transparency	2.10	0.14
gender	10.37	0.001*
grammaticality:transparency	10.06	0.002*
grammaticality:gender	7.85	0.005*
transparency:gender	0.32	0.57
grammaticality:transparency:gender	2.18	0.14
*R*^2^ Marginal = 0.005; *R*^2^ Conditional = 0.28		

800 to 900 ms	Chisq	*p*-value
grammaticality	9.60	0.002*
transparency	1.71	0.19
gender	6.52	0.01*
grammaticality:transparency	15.45	<0.001**
grammaticality:gender	13.58	<0.001**
transparency:gender	1.80	0.18
grammaticality:transparency:gender	0.16	0.69
*R*^2^ Marginal = 0.005; *R*^2^ Conditional = 0.19		

*p < 0.01, **p < 0.001 (Bonferroni correction applied).

Starting, then, with the later positivity results, these effects not only indicate a qualitatively similar processing of gender as evidenced in the previous literature for homeland natives as well as advanced L2 Spanish learners, they also indicate that HSs show increased sensitivity to morphological regularity and markedness. The higher positivity noted as being manipulated by transparency suggests that HSs are particularly attuned to, if not reliant on the relatively regular patterns of Spanish gender agreement. This is not at all surprising when we consider that, despite both being Spanish natives, HSs get much less input and opportunities to meaningfully engage with the HL than homeland natives, both over the lifespan as well as in childhood when both types of native speakers would be forming the relevant grammatical representations and the processing strategies for them. It would seem then that quantity and quality of input distinctions between the two sets of natives are not merely responsible for observed differences in how the two assign gender at the lexical level for opaque nouns themselves—they cannot be reinforced by a regular morphophonological rule—but indeed how they process agreement for nouns in real-time when the overt rule cannot have a bootstrapping effect.

The fact that gender also has an effect means that markedness plays a distinct role, which again is unsurprising. Herein, this means that agreement mismatch errors reflecting a feature-clash was more costly for processing, yielding a more positive P600 effect. Recall that such an effect has also been found for homeland Spanish natives (Alemán Bañón and Rothman, 2016), offering further evidence that HSs processing of gender is qualitatively similar to other Spanish natives. However, given that this markedness effect is found also at the behavioral (in assignment and GJT) and electrophysiological levels (in Alemán Bañón and Rothman, 2016 it is only at the brain level) and is accompanied by the present transparency effect, we would like to interpret the whole picture as supporting the interpretation offered immediately above: HSs have qualitatively similar gender representations and processing abilities but their context/reality of acquisition and use of the HL over time makes them more sensitive to overt morphological patterns for real-time processing. Such an interpretation is well in line with the argumentation offered in recent behavioral processing studies such as in Di Pisa et al. (2022), where Italian HSs showed similar significant effects for both transparency and markedness despite these same effects not being replicated in the homeland Italian and sequential L2 learner comparison groups.

## 4. General discussion

The present study investigated the qualitative nature of grammatical gender processing in Spanish as an HL. More specifically, the study aimed to examine whether transparency of the gender cue on the head noun, markedness and/or an interaction between the two would modulate the observed ERP components. Having unpacked the significance of what was observed already in the *Results* section, herein we offer a more general discussion by means of returning to the two research questions offered in the *Introduction*.

Question 1: What are the ERP signatures of grammatical gender processing in Spanish as a Heritage Language?

Overall, the results of our study revealed clear evidence that (our) HSs of Spanish show a P600 effect while processing gender agreement violations. Importantly, such results are consistent with ERPs studies examining the same property in homeland natives and advanced adult L2 learners. In other words, the present HSs, as a group, are sensitive to grammatical gender violations and process them in a qualitatively similar way to homeland natives. Thus, we interpret the present evidence as HSs having the same underlying grammar in the relevant sense, that is, a system of (morpho)syntactic grammatical gender that is equivalent to other Spanish native speakers.

However, this does not mean that the present HSs show exactly the same effects that have been reported in the homeland native speaker literature. For example, while our data show the classic P600 effect, there was no evidence that the P600 was preceded by a LAN. As discussed in the literature review, many, but crucially not all, studies with homeland natives have shown these two signatures to co-occur. And so, the absence of this co-occurrence is not terribly noteworthy or needing of too much discussion, not least as our methodology follows rather closely that of Alemán Bañón and Rothman (2016) and Alemán Bañón et al. (2017), two studies where the LAN did not accompany the P600 (see Alemán Bañón and Rothman, 2016 for why they concluded this was the case). However, there is a novelty to our data that is worthy of serious consideration, namely, the aggregate biphasic N400-P600. Indeed, this is not attested in the homeland Spanish natives’ literature. While this co-occurrence, to our knowledge, is also not reported in the non-native L2 literature either, it is worth noting that in addition to studies with advanced L2 speakers often showing a P600 for gender agreement violations, studies with lower levels of L2 proficiency have shown an N400 for such violations (see Alemán Bañón et al., 2018 for review and discussion the N400 to P600 shift as a function of proficiency). And so, an N400 effect is not unattested for gender processing in the bilingual literature. Yet, in the case of L2 acquisition, not least as it seems to be indicative of lower proficiency, such an effect might signal qualitatively distinct processing related to particularly unstable representations or the lack of a qualitatively similar one. In other words, gender in lower proficiency might not yet be stabilized at the lexical level or might be absent such that the noted effect is more a reflection of the L2 learners doing something else entirely, for example, noting the breakdown of the morphophonological pattern matching.

We reject *a priori* the latter applying to our HSs for several reasons. Firstly, recall that the P600 co-occurs, suggesting that grammatical integration/reanalysis is taking place. Second, if this were applicable, we might expect this only—or at least more significantly—for transparent nouns where the final vowel should match the inflection downstream on the adjective. This is not the case, however. Conversely, if the N400-P600 biphasic effect were only found for opaque nouns, we might be inclined to interpret it as evidence for the former account related to unstable lexical representations since the N400 often occurs in the context of difficulties in lexical processing. Under such a scenario, this explanation would seem reasonable since when the morphology is opaque one is strictly reliant on the lexical representation of gender—no morphophonological rule *per se* can apply. If our HSs have unstable gender assignment representations for such nouns, they might, then, have greater difficulty that would be reflected at the lexical level and thus demonstrable via an N400 effect for such nouns only. Yet, this is also not the case, the biphasic pattern is not conditioned by the transparency of the head noun. In our view, we do not have convincing EEG-related evidence or behavioral evidence for that matter to suggest that the present HSs have unstable gender assignment representations for opaque nouns *per se*. While claiming so is a reasonable argument to make for L2 learners in the process of language acquisition, as has been done with supporting evidence in the above cited work, one needs to be considered when applying the same logic to the case of HSs precisely because adult HSs are not in at intermediary stage of acquisition when tested. While we do have behavioral evidence showing the HSs are less accurate with feminine opaque nouns, this is not unexpected and, crucially, one need not resort to claims of unstable representations to make sense of it. To the extent that masculine is the default, we would expect what our data bear out: considerably higher accuracy for masculine opaque nouns along with degraded accuracy with feminine counterparts. It is important to make clear that low accuracy and instability are not the same thing, the former does not (necessarily) entail the latter as the source. Instability would appropriately apply if data were to show indeterminacy in gender assignment, for example, if HSs had had to provide the appropriate article for given nouns in the assignment task multiple times and showed inconsistency in doing so. If this were significantly more the case for opaque nouns in general or only for opaque feminine, appealing to instability in their system would have some empirical grounding. However, since our assignment task only had one instance for each item given the sheer size of the list of nouns, it is possible that for 34% of normatively-speaking “feminine” opaque nouns for which a masculine article was provided, HSs have different, yet stable masculine representations. If so, instability to describe this would be descriptively inaccurate. Rather, at most, it would reflect instances of misassignment, although we would be reticent to label it as such given that misassignment (accuracy for that matter) is based on differences to a consensus of non-bilingual norms. Simply put, we have no direct evidence, or at least not the right type of evidence in the present methodology, to suggest unstable gender representations. That the pattern of performance, however, follows what one would expect based on transparency/markedness considerations and reinforces the importance of them in HS contexts, where input and domains of use are often reduced compared to other early naturalistic acquirers of the language.

We would like to consider, then, two explanations for the HS biphasic effect, not mutually exclusive to each other and both of which require further, future work to best (dis)confirm. The first thing to consider emerges on the coattails of a series of ERP studies addressing the universality and variability behind the neural correlates of morphosyntactic processing (in homeland natives and non-dominant bilinguals alike), where N400s have also been found to be elicited in response to grammatical violations for which P600s are (in theory) expected (*cf.* Pakulak and Neville, 2010; Tanner and Van Hell, 2014; Grey and van Hell, 2017; Kim et al., 2018; Tanner, 2019; Grey, 2023). Findings from these studies have revealed intrinsic and dynamic individual-level variability, both between and within-subjects, present in both L1 and L2 processing during online sentence comprehension. Results show that even when the P600 emerges (and dominates) after grand averaging, there is a need to move away from the traditional interpretation that the P600 alone indexes morphosyntactic violations. Rather, an individual-difference framework that accounts for variability in language processing routes and provides the space to examine its relationship with other learner-internal and external factors should be considered. Thus, we do not want to dismiss the possibility that, in our pool of HSs, there are enough individuals—essentially a balance between the two types—who take an N400 and a P600 route at the individual level that then in our grand averaging conserves both emerging and leaving the impression that there is a true biphasic N400-P600 group effect. If on the right track, then, it would be the case that the biphasic pattern observed is not representative of any (or very few) HS individuals. At present, we do not have a large enough sample to meaningfully unpack this. Thus, we leave testing this possibility to a future date when we have enough participants, as in Tanner and Van Hell (2014) or Grey (2023), to see if indeed the N400-P600 pattern is truly representative of all our HSs or, if, rather at least some of this pattern is more reflective of a split in individual performance along a continuum whereby some might be more N400-dominant, while others might be more P600-dominant during online gender processing. While it would be worthwhile to pursue the N400-P600 continuum for the present and other, independent reasons in HS processing, we should acknowledge a few things. With a larger sample, this biphasic pattern might not be upheld. Since both signatures co-occur presently at the aggregate level, if it is the case that the biphasic pattern is not truly descriptive of the group’s individuals, then it would need to be the case that there is a near equal amount of N400 and P600 dominant processors for both to survive the grand averaging. In this case, we would want to know if our present distribution is, then, merely happenstance or what variables might explain which (and why) individuals fall more and less into one or the other camp. In any case, with more participants the balance might tip in one direction or the other such that the aggregate no longer shows a biphasic grand averaging. Nevertheless, data such as the present underscore the utility and need for doing individual-level EEG analyses when possible.

For now, however, let us offer/consider some potential insights into what we think would underlie a true biphasic N400-P600 response, whether this truly reflects all individuals of the HS aggregate or in the case, it turns out to be only some of them along a continuum as suggested above. As discussed already, our results lead strongly to the conclusion that HSs are quite sensitive to overt morphology. The present study provides converging evidence from both brain (ERPs) and behavior (agreement judgment and assignment) in this respect. While homeland Spanish natives have also been shown to be sensitive to markedness via ERP testing, for example, the degree of sensitivity of the present HSs to both markedness and crucially transparency offline and online not only seems profound but echoes what recent studies have shown for Italian HSs (Di Pisa et al., 2022), where it has been concluded that HSs are likely more sensitive to functional morphology as a compensatory strategy for the very real quantitative differences that their reality of input exposure and opportunities for use imparts. To the extent that HSs are indeed more sensitive to morphology, then the biphasic N400-P600 pattern we observed should not be surprising. Their grammatical representations for gender are qualitatively the same as other types of Spanish native speakers, hence the P600 effect indexes errors in agreement while the N400 itself indexes their enhanced sensitivity to morphology since the locus to establish agreement is lexical in nature at the same time: the gender feature’s lexicalization in the mental representation of the head noun. Such an account is not mutually exclusive, as we alluded to there being individual differences. To the extent that all HSs or only some HSs show this novel pattern—unattested in homeland natives and L2 speakers alike—the above might underlie why this is so. If it turns out that, indeed this is only true for some HSs, future research would want to pursue what exponents of particular HS experiences with their HL give rise to their (and not others’) greater sensitivity to morphology in syntactic processing. We leave this, then, also as an open question for future research with larger populations done in tandem with teasing out the applicability of this pattern to the many or the few.

Question 2: Do we find evidence of neurophysiological signatures related to the processing of grammatical gender being modulated by various aspects of overt morphology (i.e., transparency and markedness)?

While we have addressed Question 2 in detail above, we summarize the main findings further. Our data, both behavioral and ERP, indicated that our HSs show increased sensitivity to both morphological transparency and markedness when processing gender agreement (violations). While HSs displayed the typical P600 signature for gender processing, indicating that their grammars have qualitatively similar and robust representations for gender, the fact that this typical signature is accompanied by a not-so-typical (in this domain) N400 as well as the fact that their brain responses are significantly conditioned by transparency and markedness effects lead us to the conclusion that morphology has particularly high weighting for this set of natives. We argued that this is likely to be the case because the typical context of HSs involves reduced input and opportunity to use the HL in both real and apparent timeframes: as children when they were stabilizing their HL grammar and over time as they develop. It should come as no surprise that such a reality would have consequences for HL grammars, especially at the level of processing where we believe innovations in our HSs’ performances lie—implicitly compared to what homelands have been shown to do. The syntax of gender seems to be well established and in place, whatever input our HSs have had was enough to instantiate this into their HL grammars. Yet, in light of the reduced nature of their exposure and opportunities for engaging with Spanish over time as their dominance shifted toward the majority language, their systems have become optimized to rely more on morphological/morphophonological patterns. We interpret these results, then, in the most positive of lights: the present HS data can be understood as an embodiment of “doing more with less.”

## Data availability statement

The datasets presented in this study can be found in online repositories. The names of the repository/repositories and accession number(s) can be found at: https://osf.io/57gac/?view_only=362c66d50dd5437cb0696c4116b7a097.

## Ethics statement

The studies involving human participants were reviewed and approved by Internal Review Board (IRB) at the University of Florida. The patients/participants provided their written informed consent to participate in this study.

## Author contributions

AL, ER, and JR designed the study. AL, CR, MN, CL-R, and YR performed the data collection. AL, MK, CR, and ER processed and analyzed the data. AL, MK, CR, MN, CL-R, YR, ER, and JR contributed to the analyses, writing of the paper, and its revisions. All authors contributed to the article and approved the submitted version.

## Funding

This work was done as part of the Heritage-Bilingual Linguistic Proficiency in the Native Grammar (HeLPiNG): Charting and Explaining Differences grant, generously funded by the Tromsø Forskningsstiftelse (TFS) foundation (2019–2023).

## Conflict of interest

The authors declare that the research was conducted in the absence of any commercial or financial relationships that could be construed as a potential conflict of interest.

## Publisher’s note

All claims expressed in this article are solely those of the authors and do not necessarily represent those of their affiliated organizations, or those of the publisher, the editors and the reviewers. Any product that may be evaluated in this article, or claim that may be made by its manufacturer, is not guaranteed or endorsed by the publisher.

## Footnotes

^1^During peer-review, two reviewers expressed concerns about the inclusion of these four participants due to potential gender interference effects from Portuguese. To ensure transparency and reproducibility, we clarify our rationale for retaining them in our final dataset. Our analysis of their behavioral performance showed no significant differences compared to the remaining cohort, indicating no evidence of potential gender interference effects from Portuguese. Notably, these individuals were heritage speakers of both Spanish and Portuguese, underscoring the fact that some speakers have multiple home languages that differ from the majority societal language. Thus, we believed that excluding these participants without evidence of interference would overlook the diverse reality of Spanish heritage speakers, therefore, they remained part of our final dataset and data analyses.

^2^ https://osf.io/57gac/?view_only=f08cc9da3a384e2ba1995f34980c0890

^3^As suggested by one of our reviewers, we also ran an analysis using the d’prime score from the Signal Detection Theory (Heeger and Landy, 1997), taking into account hits, misses, false alarms, and correct rejection rates. We calculated the d-prime values subset by Transparency (transparent vs. opaque) and Gender (feminine vs. masculine). Overall, the d-prime analyses revealed a similar output to the model we ran for behavioral acceptance data: there was a significant effect of Transparency and Gender—with transparent having higher d’prime score than opaque and masculine having higher d’prime score than feminine. Similar to the acceptance model reported on this manuscript, we found no significant interaction between Transparency and Gender. Please refer to the R Markdown detailed analysis script available on our public OSF site: https://osf.io/57gac/?view_only=f08cc9da3a384e2ba1995f34980c0890.

## References

[ref1] Afonso O. Domínguez A. Álvarez C. J. Morales D. (2014). Sublexical and lexico-syntactic factors in gender access in Spanish. J. Psycholinguist. Res. 43, 13–25. doi: 10.1007/s10936-012-9236-0, PMID: 23377903

[ref2] Alarcón I. V. (2011). Spanish gender agreement under complete and incomplete acquisition: early and late bilinguals' linguistic behavior within the noun phrase. Biling. Lang. Congn. 14, 332–350. doi: 10.1017/s1366728910000222

[ref3] Alemán Bañón J. Fiorentino R. Gabriele A. (2018). Using event-related potentials to track morphosyntactic development in second language learners: the processing of number and gender agreement in Spanish. PLoS One 13:e0200791. doi: 10.1371/journal.pone.0200791 30052686 PMC6063416

[ref4] Alemán Bañón J. Miller D. Rothman J. (2017). Morphological variability in second language learners: an examination of electrophysiological and production data. J. Exp. Psychol. Learn. Mem. Cogn. 43:1509. doi: 10.1037/xlm0000394 28333508

[ref5] Alemán Bañón J. Rothman J. (2016). The role of morphological markedness in the processing of number and gender agreement in Spanish: an event-related potential investigation. Language, Cognition and Neuroscience 31, 1273–1298. doi: 10.1080/23273798.2016.1218032

[ref6] Anderson N. J. (1999). Exploring second language reading: Issues and strategies (pp. 53–56). Boston, MA: Heinle & Heinle.

[ref7] Antón-Méndez I. Nicol J. L. Garrett M. F. (2002). The relation between gender and number agreement processing. Syntax 5, 1–25. doi: 10.1111/1467-9612.00045

[ref8] Arias-Trejo N. Alva E. A. (2013). Early Spanish grammatical gender bootstrapping: learning nouns through adjectives. Dev. Psychol. 49, 1308–1318. doi: 10.1037/a0029778 22889397

[ref9] Baayen R. H. Davidson D. J. Bates D. M. (2008). Mixed-effects modeling with crossed random effects for subjects and items. J. Mem. Lang. 59, 390–412. doi: 10.1016/j.jml.2007.12.005

[ref10] Barber H. Carreiras M. (2005). Grammatical gender and number agreement in Spanish: an ERP comparison. J. Cogn. Neurosci. 17, 137–153. doi: 10.1162/0898929052880101 15701245

[ref11] Battistella E. L. (1990). Markedness: the evaluative superstructure of language. Albany, NY: SUNY Press.

[ref13] Bayram F. Pisa G. Rothman J. Slabakova R. (2021). “Current trends and emerging methodologies in charting heritage language grammars,” in The Cambridge handbook of heritage languages and linguistics. eds. Montrul S. Polinsky M. (Cambridge: Cambridge University Press), 545–578.

[ref14] Brouwer H. Fitz H. Hoeks J. (2012). Getting real about semantic illusions: rethinking the functional role of the P600 in language comprehension. Brain Res. 1446, 127–143. doi: 10.1016/j.brainres.2012.01.055 22361114

[ref15] Caffarra S. Barber H. A. (2015). Does the ending matter? The role of gender-to-ending consistency in sentence reading. Brain Res. 1605, 83–92. doi: 10.1016/j.brainres.2015.02.018 25701716

[ref16] Caffarra S. Barber H. Molinaro N. Carreiras M. (2017). When the end matters: influence of gender cues during agreement computation in bilinguals. Lang. Cogn. Neurosci. 32, 1069–1085. doi: 10.1080/23273798.2017.1283426

[ref17] Corbett G. G. (1991). Gender. Cambridge, UK: Cambridge University Press.

[ref18] Coulson S. King J. W. Kutas M. (1998). Expect the unexpected: event-related brain response to morphosyntactic violations. Lang. Cogn. Process. 13, 21–58. doi: 10.1080/016909698386582

[ref19] Cowper E. (2005). The geometry of interpretable features: Infl in English and Spanish. Language 81, 10–46. doi: 10.1353/lan.2005.0012

[ref20] Cuetos F. Glez-Nosti M. Barbón A. Brysbaert M. (2012). SUBTLEX-ESP: Spanish word frequencies based on film subtitles. Psicológica 33, 133–143.

[ref21] De la Cruz Cabanillas I. Martínez C. T. Prados M. D. Redondo E. C. (2007). English loanwords in Spanish computer language. Engl. Specif. Purp. 26, 52–78. doi: 10.1016/j.esp.2005.06.002

[ref22] Di Pisa G. Kubota M. Rothman J. Marinis T. (2022). Effects of markedness in gender processing in Italian as a heritage language: a speed accuracy tradeoff. Front. Psychol. 13:965885. doi: 10.3389/fpsyg.2022.965885, PMID: 36312081 PMC9596986

[ref23] Friederici A. D. (2002). Towards a neural basis of auditory sentence processing. Trends Cogn. Sci. 6, 78–84. doi: 10.1016/s1364-6613(00)01839-8 15866191

[ref24] Fuchs Z. (2021). Facilitative use of grammatical gender in heritage Spanish. Linguist. Approach. Biling. 12, 845–871. doi: 10.1075/lab.20024.fuc

[ref25] Fuchs Z. (2022). Eyetracking evidence for heritage speakers’ access to abstract syntactic agreement features in real-time processing. Front. Psychol. 13:960376. doi: 10.3389/fpsyg.2022.960376 36248451 PMC9562099

[ref26] Fuchs Z. Polinsky M. Scontras G. (2015). The differential representation of number and gender in Spanish. Linguist. Rev. 32, 703–737. doi: 10.1515/tlr-2015-0008

[ref27] Gabriele A. Fiorentino R. Bañón J. A. (2013). Examining second language development using event-related potentials: a cross-sectional study on the processing of gender and number agreement. Linguist. Approach. Biling. 3, 213–232. doi: 10.1075/lab.3.2.04gab

[ref28] Gathercole S. E. (2002). “Memory development during the childhood years,” in Handbook of memory disorders. eds. Baddeley A. D. Kopelman M. D. Wilson B. A.. 2nd ed (Chichester, UK: John Wiley & Sons, Ltd), 475–500.

[ref29] Gathercole V. C. M. Stadthagen-González H. Parafita Couto M. C. De Mulder H. N. Pérez-Tattam R. S. Bosma E.. (2022). “Moveable figures and grounds: making the case for the dual nature of motion events as events of motion and change of state” in Developing language and literacy (Cham: Springer), 129–172.

[ref30] Gathercole V. C. M. Thomas E. M. (2005). “Minority language survival: input factors influencing the acquisition of Welsh” in Proceedings of the 4th international symposium on bilingualism (Somerville, MA: Cascadilla Press), 852–874.

[ref31] Grey S. (2023). Variability in native and nonnative language: An ERP study of semantic and grammar processing. Stud. Second Lang. Acquis. 45, 137–166. doi: 10.1017/S0272263122000055

[ref32] Grey S. van Hell J. G. (2017). Foreign-accented speaker identity affects neural correlates of language comprehension. J. Neurolinguistics 42, 93–108. doi: 10.1016/j.jneuroling.2016.12.001

[ref33] Gunter T. C. Friederici A. D. Schriefers H. (2000). Syntactic gender and semantic expectancy: ERPs reveal early autonomy and late interaction. J. Cogn. Neurosci. 12, 556–568. doi: 10.1162/089892900562336 10936910

[ref34] Harris J. W. (1991). The exponence of gender in Spanish. Linguist. Inquiry 22, 27–62.

[ref35] Heeger D. Landy M. (1997). Signal detection theory. Stanford, CA: Dept. Psych., Stanford Univ..

[ref36] Hurr E. Lopez Otero J. C. Sanchez L. (2020). Gender agreement and assignment in Spanish heritage speakers: does frequency matter? Languages 5:48. doi: 10.3390/languages5040048

[ref37] Izura C. Cuetos F. Brysbaert M. (2014). Lextale-Esp: a test to rapidly and efficiently assess the Spanish vocabulary size. Psicológica 35, 49–66. doi: 10.14691/CPPJ.22.4.19

[ref38] Kaan E. (2007). Event-related potentials and language processing: a brief overview. Lang. Linguist. Compass 1, 571–591. doi: 10.1111/j.1749-818X.2007.00037.x

[ref39] Kaan E. Grüter T. (Eds.). (2021). Prediction in second language processing and learning. Amsterdam, Netherlands: John Benjamins Publishing Company.

[ref40] Kim A. E. Oines L. Miyake A. (2018). Individual differences in verbal working memory underlie a tradeoff between semantic and structural processing difficulty during language comprehension: an ERP investigation. J. Exp. Psychol. Learn. Mem. Cogn. 44, 406–420. doi: 10.1037/xlm0000457 28933902

[ref41] Kupisch T. Akpınar D. Stöhr A. (2013). Gender assignment and gender agreement in adult bilinguals and second language learners of French. Linguist. Approach. Biling. 3, 150–179. doi: 10.1075/lab.3.2.02kup

[ref42] Kupisch T. Rothman J. (2016). Terminology matters! Why difference is not incompleteness and how early child bilinguals are heritage speakers. Int. J. Biling. 22, 564–582. doi: 10.1177/1367006916654355

[ref43] Kutas M. Federmeier K. D. (2011). Thirty years and counting: finding meaning in the N400 component of the event relatedevent-related brain potential (ERP). Annu. Rev. Psychol. 62:621. doi: 10.1146/annurev.psych.093008.131123 20809790 PMC4052444

[ref44] Lenth R. V. (2022). Emmeans: estimated marginal means, aka least-squares means. R package version 1.7.5. Available at: https://CRAN.R-project.org/package=emmeans

[ref45] Lew-Williams C. Fernald A. (2007). Young children learning Spanish make rapid use of grammatical gender in spoken word recognition. Psychol. Sci. 18, 193–198. doi: 10.1111/j.1467-9280.2007.01873.x 17444909 PMC3206966

[ref46] Li P. Zhang F. Yu A. Zhao X. (2020). Language history questionnaire (LHQ3): an enhanced tool for assessing multilingual experience. Biling. Lang. Congn. 23, 938–944. doi: 10.1017/s1366728918001153

[ref47] Lipski J. (1993). “Creoloid phenomena in the Spanish of transitional bilinguals” in Spanish in the United States. eds. Roca A. Lipski J. M. (Berlin, Germany: De Gruyter Mouton), 155–173. doi: 10.1515/9783110804973

[ref48] López Ornat S. Fernández A. Gallo P. Mariscal S. (1994). La adquisición de la Lengua española [the acquisition of the Spanish language]. Madrid, Spain:Edinumen.

[ref49] López-Ornat S. (1997). “What lies in between a pre-grammatical and a grammatical representation? Evidence on nominal and verbal form-function mapping in Spanish from 1;7 to 2;1,” in Contemporary perspectives on the acquisition of Spanish. eds. Pérez-Leroux A. T. Glass W. (Sommerville, MA: Cascadilla Press), 3–20.

[ref001] Luck S. J. Kappenman E. S. (Eds.). (2011). The Oxford handbook of event-related potential components. Oxford university press.

[ref50] Mariscal S. (2009). Early acquisition of gender agreement in the Spanish noun phrase: starting small. J. Child Lang. 36, 143–171. doi: 10.1017/s0305000908008908 18761756

[ref51] Martohardjono G. Phillips I. Madsen C. N. II Schwartz R. G. (2017). “Cross-linguistic influence in bilingual processing: an ERP study.” in *Proceedings of the 41st Boston University Conference on Language Development* (Vol. 2, pp. 452–465). Somerville, MA: Cascadilla Press.

[ref52] Molinaro N. Barber H. A. Carreiras M. (2011). Grammatical agreement processing in reading: ERP findings and future directions. Cortex 47, 908–930. doi: 10.1016/j.cortex.2011.02.019 21458791

[ref53] Montrul S. (2004). Subject and object expression in Spanish heritage speakers: a case of morphosyntactic convergence. Biling. Lang. Congn. 7, 125–142. doi: 10.1017/S1366728904001464

[ref002] Montrul S. (2011). Introduction: The linguistic competence of heritage speakers. Stud. Second Lang. Acquis. 33, 155–161. doi: 10.1017/S0272263110000719

[ref54] Montrul S. (2016). Losing your case? Dative experiencers in Mexican Spanish and heritage speakers in the United States. Adv. Spanish Heritage Lang. 49:126. doi: 10.1075/sibil.49.06mon

[ref55] Montrul S. Foote R. Perpiñán S. (2008). Gender agreement in adult second language learners and Spanish heritage speakers: the effects of age and context of acquisition. Lang. Learn. 58, 503–553. doi: 10.1111/j.1467-9922.2008.00449.x

[ref56] O'Rourke P. L. Van Petten C. (2011). Morphological agreement at a distance: dissociation between early and late components of the event-related brain potential. Brain Res. 1392, 62–79. doi: 10.1016/j.brainres.2011.03.071 21466794

[ref57] Osterhout L. Mobley L. A. (1995). Event-related brain potentials elicited by failure to agree. J. Mem. Lang. 34, 739–773. doi: 10.1006/jmla.1995.1033

[ref58] Pakulak E. Neville H. J. (2010). Proficiency differences in syntactic processing of monolingual native speakers indexed by event-related potentials. J. Cogn. Neurosci. 22, 2728–2744. doi: 10.1162/jocn.2009.21393 19925188 PMC2891257

[ref59] Pérez-Pereira M. (1991). The acquisition of gender: what Spanish children tell us. J. Child Lang. 18, 571–590. doi: 10.1017/s0305000900011259 1761614

[ref60] Phillips C. Ehrenhofer L. (2015). The role of language processing in language acquisition. Linguist Approach Biling 5, 409–453. doi: 10.1075/lab.5.4.01phi

[ref61] Polinsky M. (2008). Gender under incomplete acquisition: heritage speakers’ knowledge of noun categorization. Herit. Lang. J. 6, 40–71. doi: 10.46538/hlj.6.1.3

[ref62] Polinsky M. (2018). Heritage languages and their speakers. Cambridge, UK: Cambridge University Press.

[ref63] Polinsky M. Scontras G. (2020). Understanding heritage languages. Biling. Lang. Congn. 23, 4–20. doi: 10.1017/S1366728919000245

[ref64] Psychology Software Tools, Inc. [E-Prime 3.0]. (2016). Available at: https://support.pstnet.com/

[ref003] R Core Team. (2016). R: A Language and Environment for Statistical Computing. Vienna, Austria. Available at: https://www.R-project.org/

[ref65] Rodina Y. Westergaard M. (2017). Grammatical gender in bilingual Norwegian–Russian acquisition: the role of input and transparency. Biling. Lang. Congn. 20, 197–214. doi: 10.1017/S1366728915000668

[ref66] Rossi E. Pereira Soares S. Prystauka Y. Nakamura M. Rothman J. (2022). Riding the (brain) waves! Using neural oscillations to inform bilingualism research. Biling. Lang. Congn. 26, 202–215. doi: 10.1017/S1366728922000451

[ref67] Rothman J. (2009). Understanding the nature and outcomes of early bilingualism: romance languages as heritage languages. Int. J. Biling. 13, 155–163. doi: 10.1177/1367006909339814

[ref68] Rothman J. Bayram F. DeLuca V. Di Pisa G. Duñabeitia J. Gharibi K.. (2022). Monolingual comparative normativity in bilingualism research is out of “control”: arguments and alternatives. Appl. Psycholinguist., 1–14. doi: 10.1017/S0142716422000315

[ref69] Sadek C. S. (1975). Theoretical basis for the development of the language arts curriculum in bilingual programs.

[ref70] Sassenhagen J. Bornkessel-Schlesewsky I. (2015). The P600 as a correlate of ventral attention network reorientation. Cortex 66, A3–A20. doi: 10.1016/j.cortex.2014.12.019 25791606

[ref71] Scontras G. Polinsky M. Fuchs Z. (2018). In support of representational economy: agreement in heritage Spanish. Glossa 3:1. doi: 10.5334/gjgl.164

[ref72] Singmann H. Bolker B. Westfall J. Aust F. Ben-Shachar M. S. (2022). Afex: Analysis of factorial experiments. R package version 1.1–1. Available at: https://CRAN.R-project.org/package=afex

[ref73] Tanner D. (2019). Robust neurocognitive individual differences in grammatical agreement processing: a latent variable approach. Cortex 111, 210–237. doi: 10.1016/j.cortex.2018.10.011 30508679

[ref74] Tanner D. Van Hell J. G. (2014). ERPs reveal individual differences in morphosyntactic processing. Neuropsychologia 56, 289–301. doi: 10.1016/j.neuropsychologia.2014.02.002 24530237

[ref75] Teschner R. V. Russell W. M. (1984). The gender patterns of Spanish nouns: an inverse dictionary-based analysis. Hispanic Linguist. 1, 115–132.

[ref76] Unsworth S. Argyri F. Cornips L. Hulk A. Sorace A. Tsimpli I. (2014). The role of age of onset and input in early child bilingualism in Greek and Dutch. Appl. Psycholinguist. 35, 765–805. doi: 10.1017/s0142716412000574

[ref77] Van de Meerendonk N. Kolk H. H. Chwilla D. J. Vissers C. T. W. (2009). Monitoring in language perception. Lang. Linguist. Compass 3, 1211–1224. doi: 10.1111/j.1749-818x.2009.00163.x

[ref78] Van Rijswijk R. V. (2016). The strength of a weaker first language: language production and comprehension by Turkish heritage speakers in the Netherlands. Utrecht: LOT.

[ref79] Wickham H. (2016). “Data analysis” in ggplot2 (Cham: Springer), 189–201.

